# Carotid blood flow, cardiovascular and endocrine responses during head-up tilt in patients with acute cerebrovascular diseases

**DOI:** 10.1186/2193-1801-3-191

**Published:** 2014-04-16

**Authors:** Takahiro Miyake, Takeshi Nakamura, Ken Kouda, Hiroyasu Uenishi, Yoshio Yamamoto, Shinji Kawasaki, Masami Ueno, Fumihiro Tajima

**Affiliations:** Department of Rehabilitation Medicine, Wakayama Medical University, School of Medicine, 811-1 Kimiidera, 641-8509 Wakayama City, Wakayama, Japan; Community Medical Support Center, Wakayama Medical University, 811-1 Kimiidera, 641-8509 Wakayama City, Wakayama, Japan

**Keywords:** Duplex ultrasound, Noradrenaline, Blood pressure, Stroke volume, Cardiac output

## Abstract

The purpose of this study was to define common carotid blood flow (CBF), cardiovascular and endocrine responses during head-up tilt (HUT) in patients with acute cerebrovascular diseases (CVD). In 31 male patients with acute CVD (damage of the supratentorial area) and 21 age-matched control male subjects, we measured CBF, mean blood pressure (MBP), heart rate, stroke volume and cardiac output responses before (baseline), during and after HUT. We also measured plasma levels of antidiuretic hormone, adrenaline, noradrenaline, aldosterone and plasma renin activity. After obtaining baseline measurements during 3-minutes horizontal position, HUT was performed for 5 minutes, followed by continuation of recording for 3 more minutes in the horizontal position. During HUT, CBF decreased significantly and equally in both groups. MBP did not change during HUT in both groups. The endocrine responses were also not different between the two groups. The results suggest that damage to the supratentorial area in patients with acute CVD do not alter the CBF, cardiovascular and endocrine responses. In other words, HUT does not predispose patients with acute CVD to serious falls in MBP and CBF at upright posture.

## Introduction

Cardiovascular and endocrine responses to head-up tilt (HUT) and the regulation of these responses have been investigated thoroughly in healthy subjects (Vargas et al. 1986; Laszlo et al. 2001; Geelen et al. 2002; Enishi et al. 2004). In addition, the common carotid blood flow (CBF) response to HUT in healthy people has also been analyzed previously (Yoshimoto et al. 1994; Wecht et al. 2004; Miyake et al. 2008). In general, the center that regulates the cardiovascular response during HUT is located in the medulla. At this stage, however, whether the supratentorial brain areas affect the cardiovascular response during HUT remains unclear. On the other hand, it was reported that the blood pressure (BP) response to HUT in patients with chronic cerebrovascular diseases (CVD) is different from that of healthy people (Enishi et al. 2004). In patients with chronic CVD, HUT results in immediate fall in BP and the magnitude of the initial BP reduction is greater than in healthy elderly subjects. Therefore, the BP and CBF responses to HUT in patients with acute CVD of supratentorial lesion might also be different, relative to healthy subjects. However, no study has examined the BP and CBF responses to HUT in such patients.

Orthostatic tolerance is the primary mechanism underlying gait function in humans. Rehabilitation programs often require patients to sit and stand, thus orthostatic training is important. In addition, early rehabilitation, with emphasis on sitting and standing, for CVD patients is recommended for a better clinical outcome (Adams et al. 2003; Bernhardt et al. 2008). Clinically, it is important to understand the symptoms, behavior of CBF, cardiovascular and endocrine responses during HUT in patients with acute CVD.

Plasma adrenaline and noradrenaline levels increase with activation of the central sympathetic nervous system during orthostatic stress. Therefore, plasma adrenaline and noradrenaline levels can evaluate the activity of the sympathetic nervous system. On the other hand, renin, aldosterone, and ADH are the most important hormones that regulate systemic fluid volume during orthostatic stress.

The purpose of the present study was to investigate CBF, cardiovascular and endocrine responses induced by HUT in patients with acute CVD. We also assessed abnormalities in CBF and BP responses, if any, during orthostatic stress in patients with acute CVD. For this purpose, we measured the mean blood pressure (MBP), heart rate (HR), stroke volume (SV), cardiac output (CO), plasma antidiuretic hormone (ADH), adrenaline, noradrenaline, aldosterone and plasma renin activity (PRA) responses during HUT in patients with acute CVD and healthy elderly males. In addition, the Duplex ultrasound system was used to measure CBF and the regional blood flow regulating system.

## Materials and methods

### Subjects

The study included 31 patients with acute CVD who were enrolled in a regular physical therapy program and 21 healthy elderly males as control subjects. The mean age, height, weight and body mass index (BMI) of each group are shown in Table 1. The age, height, weight and BMI of acute CVD patients were similar to those of the control subjects. The mean time between acute CVD with hemiplegia and the present study was 7.4 ± 2.8 days (±SD, range: 3–14 days). The medical condition of the patients was stable, and each patient received necessary medical treatment and passive exercise of extremities to sustain the range of motion. In addition, all study subjects underwent physical and neurological examination by neurologists, CT scan, biochemical blood tests, ECG, and chest X-ray to check for other medical problems. Patients with diabetes mellitus, ischemic heart disease, significant arrhythmias, and neurological disorders, except CVD and hypertension, were excluded. In all patients, the lesions of acute CVD were limited to the supratentorial area. Some patients used medications for hypertension. The Barthel index (Mahoney and Barthel 1965) was used to assess disability. Table 2 lists the stroke lesions confirmed by CT scan, clinical features, types of CVD, and medications used for hypertension. The control group comprised currently healthy elderly males who were not taking any medication known to affect the cardiovascular system.

**Table 1:** **Anthropometric data**

	Acute CVD	Elderly	P value
	(n = 31)	(n = 21)	
Age (years)	68.9 ± 7.9	67.3 ± 7.3	NS
Height (cm)	163.4 ± 5.5	166.4 ± 6.2	NS
Weight (kg)	61.2 ± 7.8	61.7 ± 8.4	NS
Body mass index (kg/m^2^)	22.8 ± 2.6	22.2 ± 2.5	NS

Data are mean ± SD.

NS: not significant.

**Table 2:** **Patient characteristics and type of cerebrovascular lesion**

No.	Age (yrs)	Type of lesion	Location of lesion	Hemiplegia	Day from onset	Barthel index	Medications for hypertension
1	76	Hemorrhage	putamen	Right	7	25	None
2	65	Infarction	corona radiate	Left	10	25	None
3	64	Hemorrhage	putamen	Right	6	40	ACE inhibitor, Ca^+2^ blocker
4	55	Hemorrhage	putamen	Left	6	20	ARB, Ca^+2^ blocker
5	65	Hemorrhage	putamen	Left	10	25	ARB, Ca^+2^ blocker
6	72	Hemorrhage	putamen	Right	7	35	None
7	55	Hemorrhage	putamen	Left	6	25	Ca^+2^ blocker
8	71	Hemorrhage	parietal lobe	Left	7	25	Ca^+2^ blocker
9	75	Infarction	corona radiate	Left	5	35	None
10	81	Infarction	parietal lobe	Left	7	35	None
11	58	Infarction	frontal lobe	Left	3	40	None
12	73	Infarction	corona radiate	Right	14	30	None
13	65	Infarction	frontal lobe	Right	5	25	Ca^+2^ blocker
14	68	Infarction	corona radiate	Right	4	25	ARB
15	63	Hemorrhage	putamen	Right	8	25	ARB, Ca^+2^ blocker
16	68	Infarction	parietal lobe	Right	8	20	ARB
17	62	Hemorrhage	putamen	Right	6	25	ARB
18	55	Infarction	parietal lobe	Right	11	30	None
19	83	Infarction	putamen	Left	7	25	Ca^+2^ blocker
20	75	Infarction	corona radiate	Left	4	25	None
21	66	Infarction	parietal lobe	Left	7	35	β1 blocker
22	72	Infarction	thalamus	Right	14	30	ARB
23	64	Infarction	frontal lobe	Right	5	30	None
24	80	Infarction	frontal lobe	Left	4	35	ACE inhibitor, Ca^+2^ blocker
25	73	Infarction	corona radiate	Right	7	40	ARB
26	65	Infarction	frontal lobe	Right	7	25	ARB, Ca^+2^ blocker
27	80	Infarction	corona radiate	Left	13	30	None
28	83	Infarction	frontal lobe	Right	5	30	None
29	64	Infarction	putamen	Left	10	40	ARB
30	75	Infarction	thalamus	Left	12	30	ARB, Ca^+2^ blocker
31	66	Infarction	putamen	Right	7	25	None

ACE: angiotensin converting enzyme, ARB: angiotensin receptor blocker.

Each subject gave a signed informed consent before participation, and the experimental protocol was approved by the Ethics Committee of Wakayama Medical University.

### Experimental protocol

All subjects reported to the laboratory at 4:00 pm on the experiment day. The room temperature was set at 26 ± 0.5°C and all subjects were dressed in cotton underwear. Subjects were also fitted with electrodes for impedance cardiography, and a blood pressure cuff was attached to the unaffected arm of the patients and right arm of the control subjects. The arm was rested at heart level on shelves fastened to the tilt table at any position. HR, SV, CO and CBF were monitored continuously throughout the study, while BP was measured every one minute. After a resting period of 40 min in the supine position on the tilt table, baseline measurements were taken over a 3-min period representing the control period. Subsequently, the tilt table was tilted to 60° within 30 sec and maintained in the HUT position for 5 min as the head-up period. The table was returned within 30 sec to the horizontal position and the subject remained in supine position for 3 min as the recovery period.

### Measurements

Impedance cardiography is a noninvasive, highly accurate procedure for continuous monitoring of hemodynamic variables, including HR, SV and CO. This method is useful for measuring SV changes in postural stress. HR and SV were estimated by impedance cardiography (MCO-101 Medi sens Inc, Japan), using the standard four-band electrode arrangement (Smith et al. 1970). A pair of disposable, self-adhesive tape electrodes was wound around the neck and base of the thorax at the level of the xiphisternum. A high frequency current was applied between the outer electrodes such that the signal voltage was proportional to intrathoracic tissue impedance obtained thought the inner electrodes. The SV (ml) was calculated as follows (Kubicek et al. 1970): SV = p (L/Z_0_)^2^ T (dZ/dt)_min_ where *p* is a constant (150 Ωcm), representing blood resistivity at 100 kHz, *L* is the mean distance between the inner pair of electrodes (cm); *Z*_*0*_ is the basal thoracic impedance (Ω); *(dZ/dt)*_*min*_ is the minimal rate of change of impedance (Ω/s); and *T* is the ventricular ejection time (s) obtained from the dZ/dt waveform. CO was calculated from the product of SV and HR.

Systolic blood pressures (SBP) and diastolic blood pressures (DBP) were measured at 1-min intervals by the auscultatory method using a sphygmomanometer on the arm, which was kept still and rested at heart level on the shelf of the tilt table during the head-up period. The MBP was calculated using the formula: MBP = [{(SBP - DBP)/3} + DBP].

Extracranial color-coded duplex sonography is a noninvasive, highly accurate procedure for recording hemodynamic data such as blood flow velocity and blood flow volume. The Duplex ultrasound system estimates the flow volume, representing the product of mean flow velocity and the cross-sectional area of the vessel, calculated from the static vessel diameter measured in the B-mode image at the location of the Doppler sample volume. This measurement provides both hemodynamic and morphological information related to brain perfusion. In this study, CBF was measured throughout the study by ultrasonography (Logic 500 pro Series GE Yokogawa Co, Tokyo). The probe was attached to the affected side in acute CVD patients, and to the right side in the control.

A 5.1-cm, 20-gauge intravascular over-the-needle catheter with a Luer plug was inserted into the forearm vein. Blood was drawn into ice-chilled tubes containing EDTA at 3, 8, and 11 min into the study, centrifuged (2000 rpm) for 10 min at 4°C, and 5-ml plasma aliquots were stored at -80°C. All samples were assayed for plasma ADH, adrenaline, noradrenaline, aldosterone, and PRA within 2 weeks of sampling.

### Statistical analysis

The values of each parameter during HUT and recovery period were compared with the baseline by repeated-measures analysis of variance. Multiple comparisons were made using Fisher’s protected least significant difference analysis (Snedecor and Cochran 1989). Differences between groups were examined for statistical significance using the *t*-test. *P* values less than 0.05 were accepted as significant. Values are expressed as mean ± SD.

## Results

### Symptoms during HUT

All subjects completed the HUT test and none of the patients and control subjects developed any symptoms, e.g. dizziness, lightheadedness, or syncope, during HUT.

### Changes in HR, SV, CO, MBP, and CBF during HUT

**Figure 1:**
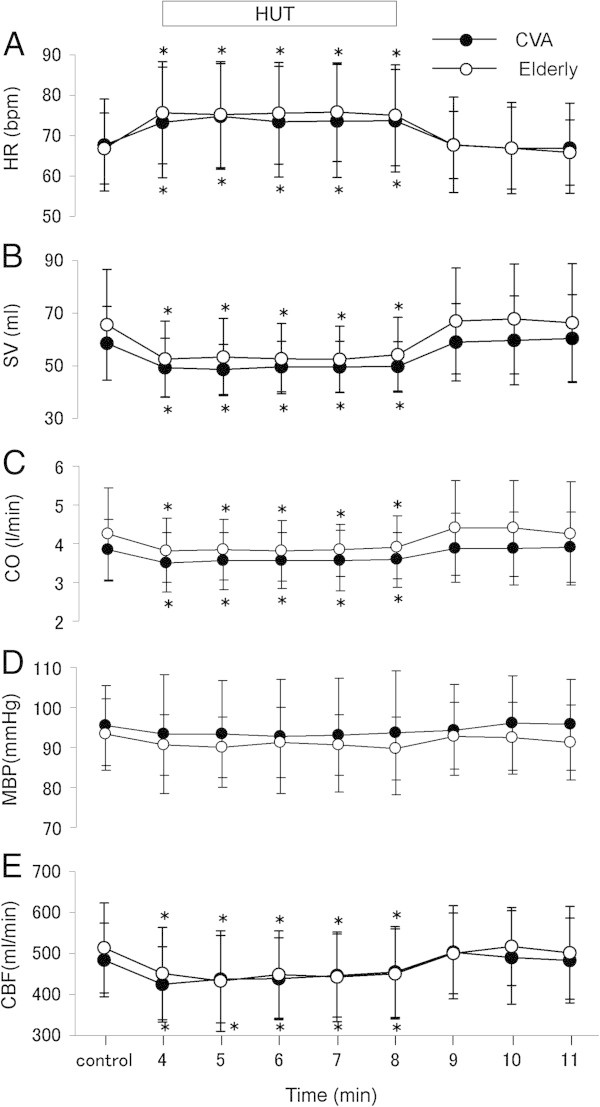
**Changes in heart rate (HR), stroke volumes (SV), cardiac output (CD), mean blood pressure (MBP) and common carotid blood flow (CBF).** **(A)** HR, **(B)** SV, **(C)** CO, **(D)** MBP, and **(E)** CBF in patients with acute CVD (n=31) and elderly subjects (n=21) at control (baseline), during a 5-minute 60° head-up tilt (HUT) and recovery. Data are mean ± SD. *P < 0.05, compared with the baseline period.

There were no differences in mean baseline values of HR (67.6 ± 11.3 and 66.8 ± 8.7 bpm, respectively), SV (58.5 ± 14.0 and 65.6 ± 21.0 ml, respectively), CO (3.8 ± 0.7 and 4.2 ± 1.2 l/min, respectively), MBP (95.4 ± 9.8 and 93.2 ± 8.9 mmHg, respectively) and CBF (484.1 ± 89.6 and 513.5 ± 109.9 ml/min, respectively) between the patients and control subjects. In both groups, HR increased significantly (p < 0.05) during HUT relative to the baseline values, and rapidly returned to the baseline levels after HUT. The magnitude of increase in HR during HUT was not different between the two groups (Figure 1A). Furthermore, SV and CO decreased significantly (p < 0.05) during HUT in both groups, and rapidly returned to control levels after HUT. The magnitudes of decrease in SV and CO during HUT were not significantly different between the two groups (Figure 1B and C). For MBP, the level remained stable during HUT in both groups, and there was no difference in MBP between the two groups throughout the entire test (Figure 1D). In both groups, CBF decreased significantly (p < 0.05) during HUT, and rapidly returned to the baseline levels after HUT. The magnitude of decrease in CBF during HUT was not significantly different between the two groups (Figure 1E).

### Endocrine responses during HUT

**Figure 2:**
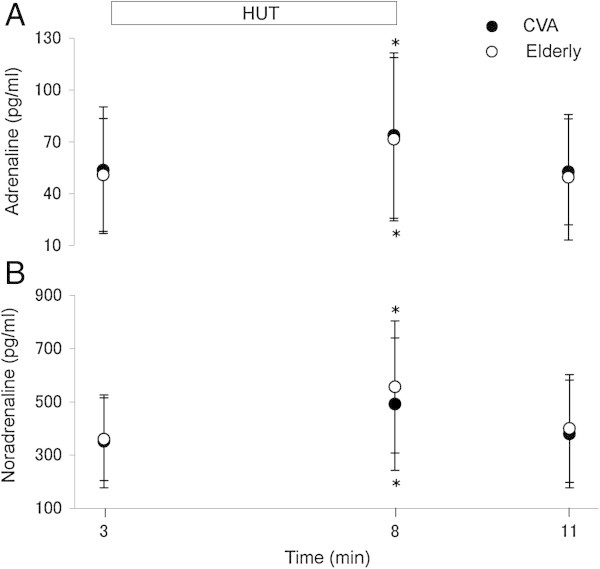
**Changes in adrenaline and noradrenaline.** Plasma levels of **(A)** adrenaline, and **(B)** noradrenaline in patients with acute CVD (n = 31) and elderly subjects (n = 21) at control (baseline), during a 5-minute 60° head-up tilt (HUT) and at recovery. Data are mean ± SD. *P < 0.05 compared with the baseline period.

**Figure 3:**
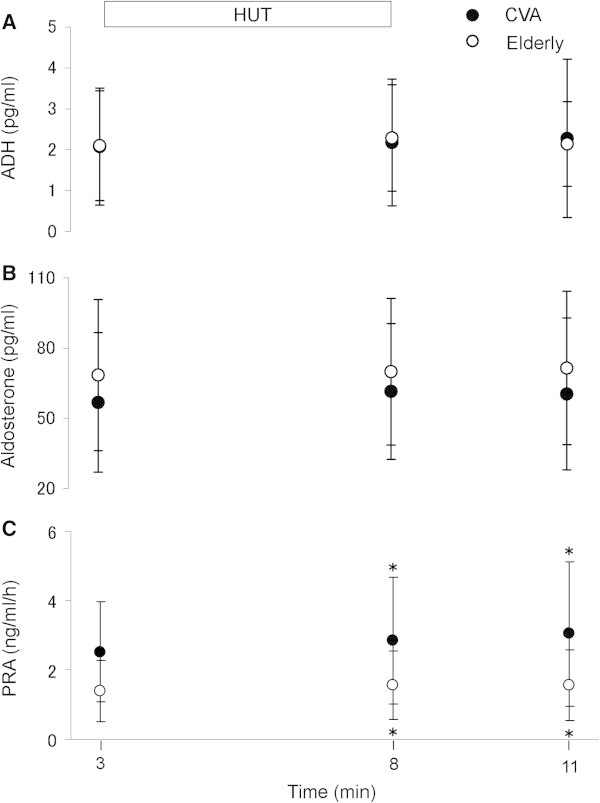
**Changes in antidiuretic hormone (ADH), aldosterone and plasma reinin activity (PRA).** Plasma levels of **(A)** plasma ADH, **(B)** aldosterone, and **(C)** PRA in patients with acute CVD (n = 31) and elderly subjects (n = 21) at control (baseline), during a 5-minute 60° head-up tilt (HUT) and at recovery. Data are mean ± SD. *P < 0.05 compared with the baseline period.

There were no differences in the baseline values of adrenaline (53.6 ± 36.5 and 50.8 ± 32.6 pg/ml, respectively), noradrenaline (351.2 ± 174.2 and 359.2 ± 155.3 pg/ml, respectively), ADH (2.0 ± 1.4 and 2.1 ± 1.3 pg/ml, respectively), aldosterone (56.8 ± 29.8 and 68.4 ± 32.3 pg/ml, respectively) and PRA (2.5 ± 1.4 and 1.4 ± 0.8 ng/ml/h, respectively) between patients and control subjects. Plasma adrenaline and noradrenaline levels increased significantly during HUT in both groups compared with the baseline values (P <0.05), and returned to baseline values during the recovery period. The magnitudes of increase in both hormones in response to HUT were not significantly different between the two groups (Figure 2A and B). HUT did not alter plasma ADH and aldosterone in both groups, and there were no differences in plasma levels of these two hormones between the two groups throughout the entire test (Figure 3A and B). PRA increased significantly during HUT in both groups compared with the baseline values (P < 0.05), and did not return to baseline values during the recovery period. However, PRA levels were not significantly different between the two groups throughout the entire test (Figure 3C).

## Discussion

The major findings of the present study were the following: 1) MBP did not change during HUT in both the patients and control subjects; 2) CBF decreased during HUT in both groups instead of retention of MBP; and 3) the magnitude of decrease in CBF during HUT was similar in the patients and control subjects. To our knowledge, this is the first study to investigate changes in CBF, neurohumoral balance and cardiovascular responses to HUT in patients with acute CVD.

During postural change from supine to upright, blood accumulates in the lower part of the body, particularly in the venous system under gravitational and hydrostatic effects (Hayashi et al. 1995). The decreased venous return is detected by cardiac and/or pulmonary mechanoreceptors and reduction of MBP activates receptors of the aortic arch and carotid sinus (Rowell 1993). In fact, the medullary vasomotor center receives pressure signals from both cardiopulmonary and arterial baroreceptors, consequently increasing HR through inactivation of vagal nerves and activation of sympathetic nerves. The vasomotor center also induces vasoconstriction of peripheral vessels by stimulating sympathetic nerve activity (Ganong 1995). Normally, this regulatory system maintains MBP during HUT in healthy subjects. In the present study, MBP was also maintained during HUT in patients with acute CVD, similar to the control subjects. Thus, it seems that the supratentorial lesions in patients with acute CVD do not alter the cardiovascular regulatory system of medullary vasomotor center during HUT. These results suggest that the cardiovascular regulatory systems to upright posture are intact in patients with acute CVD of supratentorial lesion and healthy elderly subjects.

Different to the above findings in acute CVD, previous studies reported that patients with chronic CVD showed immediate fall in BP upon HUT, and the magnitude of such fall in BP was greater than in healthy elderly subjects (Enishi et al. 2004). In the present study, however, MBP did not decrease during HUT throughout the entire test in both patients with acute CVD and the control. In our study, the average time from the onset of CVD to participation in the study was 7.4 days, compared with 33.8 months in the study of patients with chronic CVD (Enishi et al. 2004). The daily activities of CVD patients with hemiplegia are lower than healthy persons; such low activities could perhaps reduce orthostatic tolerance. Furthermore, the duration of low activities in patients with acute CVD were shorter than in patients with chronic CVD. The long duration of low activities deconditions the function of cardiovascular regulatory system (Greenleaf 1984). Therefore, the reason for the different BP response during HUT between patients with acute CVD versus those with chronic CVD could be the different duration of low physical activities. It is likely that acute CVD lesions in the supratentorial areas do not affect cardiovascular control system during HUT.

Changes in CBF are often considered representative of changes in cerebral blood flow, although the carotid artery supplies intra- and extra-cranial areas that may not be regulated in the same way (Yoshimoto et al. 1994). We have reported previously that CBF in young healthy subjects remained constant during HUT despite a decrease in CO (Miyake et al. 2008). In the present study, however, CBF and CO decreased during HUT in elderly healthy subjects. Therefore, the orthostatic tolerance to sustain cerebral blood flow might be attenuated in the elderly, and cerebral blood flow could decrease in elderly persons when they are in the upright posture. Reduced vascular compliance might be partially responsible for the decrease in CBF in upright posture in elderly subjects. On the other hand, in the present study, the magnitude of decrease in CBF during HUT in patients with acute CVD was not different from that recorded in the elderly healthy subjects. The results indicate that the cerebral blood flow regulatory system in the upright posture is also not influenced by the CVD lesions of the supratentorial areas.

Adrenaline is released as a circulating endocrine from the adrenal medulla in significant quantities in response to stimulation. The adrenal medulla is innervated by cholinergic sympathetic nerves. In contrast, noradrenaline is a neurotransmitter that leaks from sympathetic nerve terminals into plasma (Rowell 1993). It has been reported that plasma adrenaline and noradrenaline levels increase during HUT in healthy young males (Rossler et al. 1999; Ramirez-Marrero et al. 2007; Miyake et al. 2008). In our study, plasma adrenaline and noradrenaline levels also increased significantly during HUT in both groups, suggesting activation of the central sympathetic nervous system.

To adapt to the standing position for a long time, systemic circulating fluid volume and blood volume should remain constant or increase. The most important hormones known to regulate systemic fluid volume released during orthostasis are renin, angiotensins, aldosterone, and ADH (Rowell 1993). In the present study, HUT was associated with increases in PRA in both groups, but no changes in plasma levels of ADH and aldosterone were noted in both patients with acute CVD and healthy elderly subjects. We believe that the orthostatic effort in the present study was short in duration and insufficient to activate these endocrines.

This is the first report of cardiovascular and CBF responses to postural changes in patients with acute CVD. Clinically, it is important to know the changes in BP and CBF during HUT in these patients, because excess falls in BP and CBF during upright posture in these patients have a negative influence on the cerebral circulation. In the present study, upright posture did not induce larger falls in BP and CBF in patients with acute CVD compared with the healthy elderly subjects. Therefore, we believe that the head-up posture is not associated with serious derangement of the mechanisms that control the regulation of BP and CBF in patients with acute CVD.

In conclusion, the present study demonstrated a stable MBP and fall in CBF during HUT in patients with acute CVD, and that the magnitude of decrease in CBF was similar in the patient and control groups. Although the supratentorial lesions in acute CVD patients enrolled in the present study did not cover damage of the entire supratentorial area, damage to the supratentorial area in stroke patients seems less likely to alter the MBP and CBF responses induced by upright posture.
